# Fatty acid synthase-positive hepatocytes and subsequent steatosis in rat livers by irinotecan

**DOI:** 10.3892/or.2015.3814

**Published:** 2015-02-20

**Authors:** TAKEYUKI SAWANO, TAKESHI SHIMIZU, TOSHIYUKI YAMADA, NAOKI NANASHIMA, TAKUYA MIURA, SATOKO MOROHASHI, DAISUKE KUDO, FENG MAO HUI, HIROSHI KIJIMA, KENICHI HAKAMADA, SHIGEKI TSUCHIDA

**Affiliations:** 1 Departments of Gastroenterological Surgery, Hirosaki University Graduate School of Medicine, Hirosaki, Japan; 2 Departments of Biochemistry and Genome Biology, Hirosaki University Graduate School of Medicine, Hirosaki, Japan; 3 Department of Medical Technology, Hirosaki University Graduate School of Health Sciences, Hirosaki, Japan; 4 Department of Pathology and Bioscience, Hirosaki University Graduate School of Medicine, Hirosaki, Japan

**Keywords:** fatty acid synthase, liver progenitor cell, Kupffer cell, chemotherapy-associated steatohepatitis, irinotecan

## Abstract

Using a rat model, we investigated factors contributing to the pathogenesis of irinotecan-associated fatty liver disease. Male Sprague-Dawley rats were administered 200 mg/kg irinotecan by intraperitoneal injection on days 1–4, but not on days 5–7. This schedule was repeated 3 times. Rats were sacrificed 4, 18 and 25 days after the last injection, and liver steatosis was evaluated by hematoxylin and eosin (H&E) staining, microarray analysis and immunohistochemistry. Panacinar intrahepatocyte vacuoles were absent on days 4 and 25, but present on day 18, and this alteration was more prominent around the bile ducts than the central veins. Microarray analysis showed that the expression of genes involved in the synthesis of cholesterol and fatty acids was upregulated on day 4. Immunohistochemistry detected fatty acid synthase (Fasn)-strongly positive hepatocytes as well as the activation of liver progenitor cells on day 4, whereas intracellular vacuoles were evident in carbonic anhydrase 3 (CA3)-positive hepatocytes on day 18. Thus, irinotecan-induced liver steatosis was preceded by Fasn-strongly-positive hepatocytes and liver progenitor cell activation. The magnitude of the decrease in the number of Fasn-strongly positive hepatocytes between days 4 and 18 was similar to that of the increase in the number of CA3-positive hepatocytes accompanying vacuoles.

## Introduction

Colorectal cancer is one of the most commonly reported malignancies, and approximately one-third of patients with advanced colorectal cancer have liver metastasis (1). Although surgical resection is the standard treatment of colorectal cancer liver metastasis (CRLM) (2), <20% of cases are resectable at the initial diagnosis (3). Therefore, chemotherapy is frequently employed to downsize CRLM prior to surgery. New regimens, including the combination of fluorouracil and calcium folinate with irinotecan (CPT-11) (4) or oxaliplatin (5) have significantly increased tumor response rates to nearly 50% (6). These regimens followed by liver resection have been shown to improve 5-year survival rates to ~40%, which is similar to that of patients that undergo primary liver resection (7).

However, irinotecan has been associated with the development of chemotherapy-associated steatohepatitis (8) and increases mortality in subsequent hepatic surgery (9). The antitumor activity of irinotecan is dependent on the inhibition of DNA topoisomerase Ⅰ activity and is activated to SN-38, its active metabolite by carboxylesterase (10). Adverse effects associated with irinotecan such as severe neutropenia have been attributed to the inadequate inactivation of SN-38 (11). The mechanism responsible for the induction of steatohepatitis following the administration of irinotecan has not yet been elucidated. Previous clinical studies suggested that the dose or duration of the drug was not responsible (8), whereas the interval between chemotherapy and subsequent hepatic resection was correlated to the rate at which steatohepatitis was induced (12). These findings suggested that the induction of steatohepatitis may not be due to the direct toxic effects of irinotecan on hepatocytes.

Nonalcoholic steatohepatitis (NASH) is also characterized by similar pathological findings to those observed in irinotecan-associated steatohepatitis (13). This lesion shows lipid accumulation in parenchymal cells and alterations in lipid metabolism are suggested as factors involved in its pathogenesis (14,15). Kupffer cells have been shown to promote the inflammatory response associated with NASH by releasing proinflammatory mediators (16).

To explore the mechanism underlying irinotecan-associated hepatic lesions, we investigated factors contributing to their pathogenesis using an animal model. The results obtained in the present study revealed that hepatic steatosis occurred on day 18 after the last injection of irinotecan and was preceded by fatty acid synthetase strongly-positive hepatocytes and the activation of liver progenitor cells.

## Materials and methods

### Animals and animal treatment

Male Sprague-Dawley rats weighing 170–230 g, aged 6 weeks, were purchased from Clea Japan, Inc. (Shizuoka, Japan). These rats were kept under routine laboratory conditions at the animal laboratory of Hirosaki University. The rats received standard laboratory chow, had free access to food and water and were kept in a thermostatically controlled room (25°C) with a 12-h light-dark cycle. Rats were administered 200 mg/kg irinotecan by intraperitoneal injection on days 1–4, but not treated on days 5, 6 or 7. This schedule was repeated 3 times. Rats were sacrificed 4, 18 and 25 days after the last injection and livers were removed for analysis. Control rat received 2 ml/body saline intraperitoneal injections under the same schedule, and were sacrificed on day 18. Three rats were used for each time point. The present study was carried out in accordance with the Guidelines for Animal Experimentation of Hirosaki University, and all animals received humane care according to the criteria outlined in the ‘Guide for the Care and Use of Laboratory Animals’ prepared by the National Academy of Sciences and published by the National Institutes of Health (NIH publication 86–23, revised 1985).

### Microarray analysis

Total RNA was extracted from frozen liver samples with TRIzol reagent (Invitrogen, Carlsbad, CA, USA). Equal amounts of RNA from 3 individual livers were combined and 10 *μ*g of RNA was used for biotin-labeled complementary RNA (cRNA). Labeled and fragmented cRNA was subsequently hybridized to the GeneChip Rat Gene 1.0 ST Array (Affymetrix, Santa Clara, CA, USA). Labeling, hybridization, image scanning and data analysis were performed at Kurabo Industries Ltd. (Osaka, Japan).

### Quantitative real-time polymerase chain reaction (RT-PCR)

Complementary DNA (cDNA) was reverse-transcribed from 1 *μ*g of total RNA using the Omniscript RT kit (Qiagen, Tokyo, Japan). A MiniOpticon Detection System and SYBR-Green Supermix (both from Bio-Rad Laboratories, Hercules, CA, USA) were used for the quantification of specific messenger RNA (mRNA). The amplification of β-actin cDNA was performed to standardize target cDNA levels. Gene-specific primers were designed according to known rat sequences (Table I). No non-specific PCR products, as detected by melting temperature curves, were found in any case. After normalizing the expression of the target gene to the expression of β-actin, the level of the expressed mRNA in each sample was expressed relative to the control values.

### Immunohistochemistry

Tissue samples of livers were fixed in 10% neutral buffered formaldehyde and embedded in paraffin. These paraffin blocks were sliced into 4-*μ*m thick sections for hematoxylin and eosin (H&E) staining, periodic acid-Schiff (PAS) staining and immunostaining for carbonic anhydrase 3 (CA3), CD68, CD163, chemokine (C-X-C motif) ligand 9 (Cxcl9), cytokeratin 19 and fatty acid synthase (Fasn). Immunohistochemical staining was performed on deparaffinized sections using the standard avidin-biotin-peroxidase complex method with an automated immunostainer (Benchmark XT; Ventana Medical System, Tucson, AZ, USA). The primary antibodies used were: anti-CD68 antibody from AbD serotec (MCA341R) (Oxford, UK), anti-CD163 antibody from Santa Cruz Biotechnology (sc-58965) (Santa Cruz, CA, USA), anti-Cxcl9 antibody from Bioss (bs-2551R) (Boston, MA, USA), anti-cytokeratin 19 antibody from Bioworld Technology, Inc. (BS3540) (St. Louis Park, MN, USA), anti-Fasn antibody from Abcam (ab22759) (Cambridge, UK). Anti-CA3 antibodies were prepared as described by Takahata *et al* (17). Non-immune γ-globulin was used for the negative controls instead of the primary antibodies. Images were captured with an Olympus FSX100 microscope (Olympus, Tokyo, Japan). Digital images were processed with Adobe Photoshop (Adobe, San Jose, CA, USA) and ImageJ software (Wayne Rasband NIH, Bethesda, MD, USA). The percentage of intracellular vacuoles and number of Fasn-strongly-positive cells were compared between areas around the central vein (CV) and bile ducts (BD).

### Statistical analysis

Data are presented as means ± SD. Statistical evaluations were performed using the two-tailed Student’s t-test. Differences were considered to indicate a significant result with p-values <0.05.

## Results

### Induction of intrahepatocyte vacuoles after irinotecan injection

**Figure 1:**
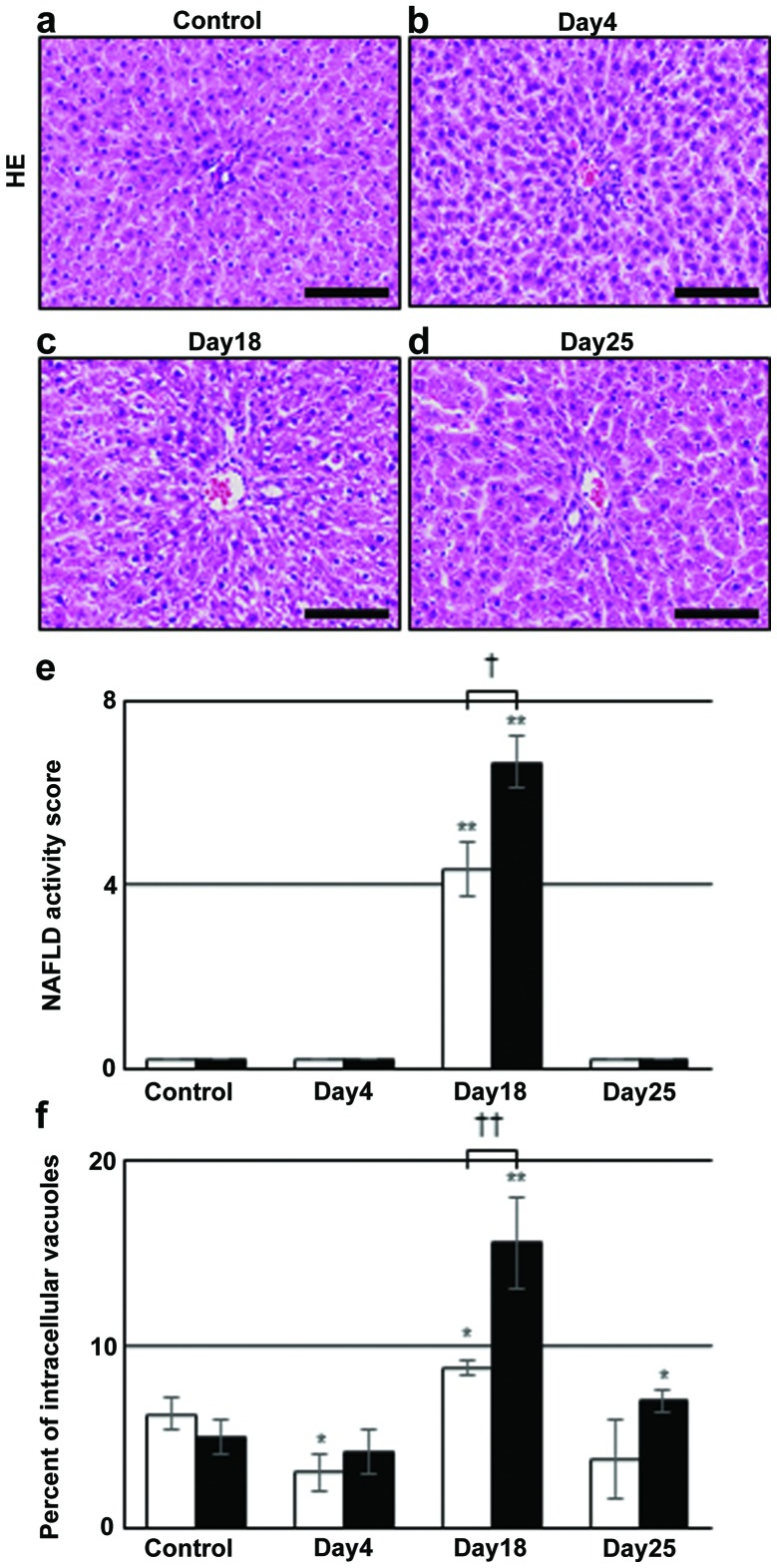
H&E staining of liver sections and the quantitative evaluation of intracellular vacuoles. (a–d) H&E staining in (a) control and irinotecan group on days (b) 4, (c) 18 and (d) 25. The data shown are from a representative preparation set and are similar to results obtained in two other sets. Intracellular vacuoles are markedly increased at day 18. Scale bars represent 100 *μ*m. (e) Nonalcoholic fatty liver disease (NAFLD) activity score on H&E-stained liver sections, depending on steatosis grade (0–3), location (0–3) and microvesicular steatosis (0–1). Open bars indicate scores around CV and closed bars those around BD. Data are the mean ± SD from 3 rats. (f) Percentage of intracellular vacuoles/unit area (0.036 mm^2^), calculated by subtracting sinusoid areas from the unstained areas of H&E-stained sections. Data are the mean ± SD from 3 rats. ^*^p<0.05 vs. control; ^**^p<0.01 vs. control (two-tailed Student’s t-test). ^†^p<0.05 and ^††^p<0.01 between values around CV and those around BD (two-tailed Student’s t-test). H&E, hematoxylin and eosin; CV, central vein; BD, bile ducts.

Liver sections from rats administered irinotecan were evaluated by H&E staining and the results are shown in Fig. 1a–d. Although no significant difference was observed between control and day 4 (Fig. 1a and b), panacinar intrahepatocyte vacuoles were detected on day 18 (Fig. 1c), and they had disappeared by day 25 (Fig. 1d). To evaluate the degree of intracellular vacuoles quantitatively, two methods were used. Firstly, we used nonalcoholic fatty liver disease (NAFLD) activity score on H&E-stained liver sections, according to Kleiner *et al* (14). The value around CV was 4.7±0.6, while that around BD was 6.7±0.6 on day 18. These values were 0 in control and days 4 and 25 in the irinotecan group (Fig. 1e). Another method used was the percentage of intracellular vacuoles per unit area calculated by subtracting sinusoid areas from the unstained areas of H&E-stained sections. Comparing with the control, this value around BD was significantly increased to 15.6±2.5 on day 18 and 7.0±0.6 on day 25 (closed bars in Fig. 1f). The value around CV was lower on day 4, but was higher at 8.8±0.4 on day 18 (open bars). These results suggested that hepatic steatosis was induced by irinotecan administration, prominent on day 18, and was more severe around BD than CV.

### Intracellular vacuole formation in CA3-positive hepatocytes

**Figure 2:**
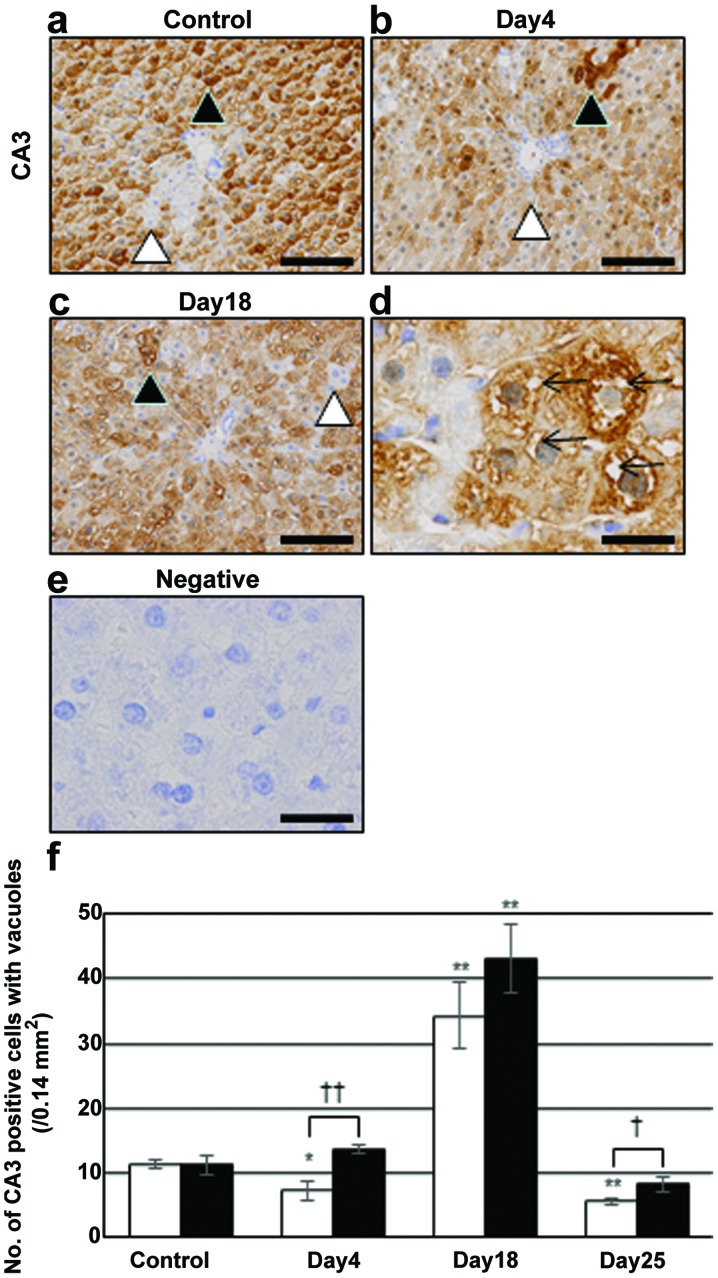
Immunostaining for CA3 in (a) control and irinotecan group on days (b) 4 and (c and d) 18. Liver sections of control group were stained with non-immune (e) γ-globulin. (d) The panel is at a higher magnification in the (c) panel. Closed and open arrowheads indicate positive and negative hepatocytes, respectively. (d) Arrows in the panel show intracellular vacuoles. The data shown are from a representative preparation set and are similar to results obtained in two other sets. (a–c) Scale bars 100 *μ*m and (d and e) 25 *μ*m. (f) Number of CA3-positive hepatocytes accompanying intracellular vacuoles. Open bars indicate the number around the CV and closed bars around the BD. Data are the mean ± SD from 3 rats. ^*^p<0.05 vs. control; ^**^p<0.01 vs. control (two-tailed Student’s t-test). ^†^p<0.05 and ^††^p<0.01 between the values for CV and for the BD (two-tailed Student’s t-test). CA3, carbonic anhydrase 3; CV, central vein; BD, bile ducts.

The expression of CA3, a marker for lipogenesis (18), was immunohistochemically evaluated to assess the presence of vacuoles in lipid-storing hepatocytes. Many hepatocytes were positive for CA3 (closed arrowheads in Fig. 2), but some cells were negative (open arrowheads). The number of CA3-positive hepatocytes was not changed after irinotecan injection. Vacuole formation in hepatocytes was easily detectable by this staining. Such vacuoles in CA3-positive cells were not observed in the control (Fig. 2a), or irinotecan group on day 4 (Fig. 2b) or 25 (data not shown), but were observed on day 18 (Fig. 2c and d). CA3-positive hepatocytes with vacuoles were mainly distributed around BD, which was consistent with the results from H&E-stained liver sections (Fig. 1).

### Enhanced expression of genes for cholesterol and fatty acid synthesis

Gene expression profiles were examined using microarray analysis in order to identify the genes responsible for inducing steatosis (Table II) (19). This revealed that the expression of genes involved in the synthesis of cholesterol and fatty acids was upregulated on day 4. The upregulated genes for the synthesis of cholesterol include A*cly*, *Hmgcr*, *Mvd*, *Sqle* and *Dhcr7*. Genes for the synthesis (*Cyp7a1*), export (*Abcg5* and *Abcc3*), and conjugation (*Ugt2a3* and *Ugt2b*) of bile acids were also upregulated. The expression of genes for the metabolism of fatty acids [*Fasn* (20), *Elovl6* and *Acss2*) was also enhanced. Sterol regulatory element-binding proteins 1 and 2 (Srebp1 and 2) have been shown to regulate the transcription of genes involved in the synthesis of cholesterol and fatty acids (21), while insulin-induced gene 1 (Insig1) and SREBP cleavage-activating protein (Scap) mediate the activation of Srebp1 and 2 (22,23). *Insig1* and *Scap* were upregulated on day 4. The expression of other transcription factors involved in lipid metabolism, *Egr1* (24) and *Nr1i3* (*CAR*) (25), was unchanged on day 4, yet upregulated by day 18. The expression of genes associated with the cell cycle and DNA replication (*Ccnb1*, *Rrm2* and *Top2a*) was downregulated on day 4, yet upregulated on days 18 and 25. A gene related to endoplasmic reticulum stress, eukaryotic translation initiation factor 2α kinase 4 (*Eif2ak4*) (26) was upregulated on days 4 and 18. The expression of Kupffer cell marker genes, except for *Cxcl9*, remained unchanged. No significant differences were observed in the expression of sinusoid and stellate cell marker genes. The expression of the liver progenitor cell marker genes, *Krt19* and *Epcam*, was upregulated on day 4. To confirm the microarray results, some mRNA levels of cholesterol, bile acid and fatty acid synthesis were quantified by RT-PCR (Table III). *Hmgcr*, *Sqle* and *Cyp7a1* mRNA were increased on day 4, as compared with control values. *Fasn* and *Acss2* mRNA levels were increased on days 4 and 18. *Egr1* mRNA was markedly increased on day 18. *Cxcl9* was downregulated on days 4 and 18.

### Appearance of Fasn-strongly-positive hepatocytes

**Figure 3:**
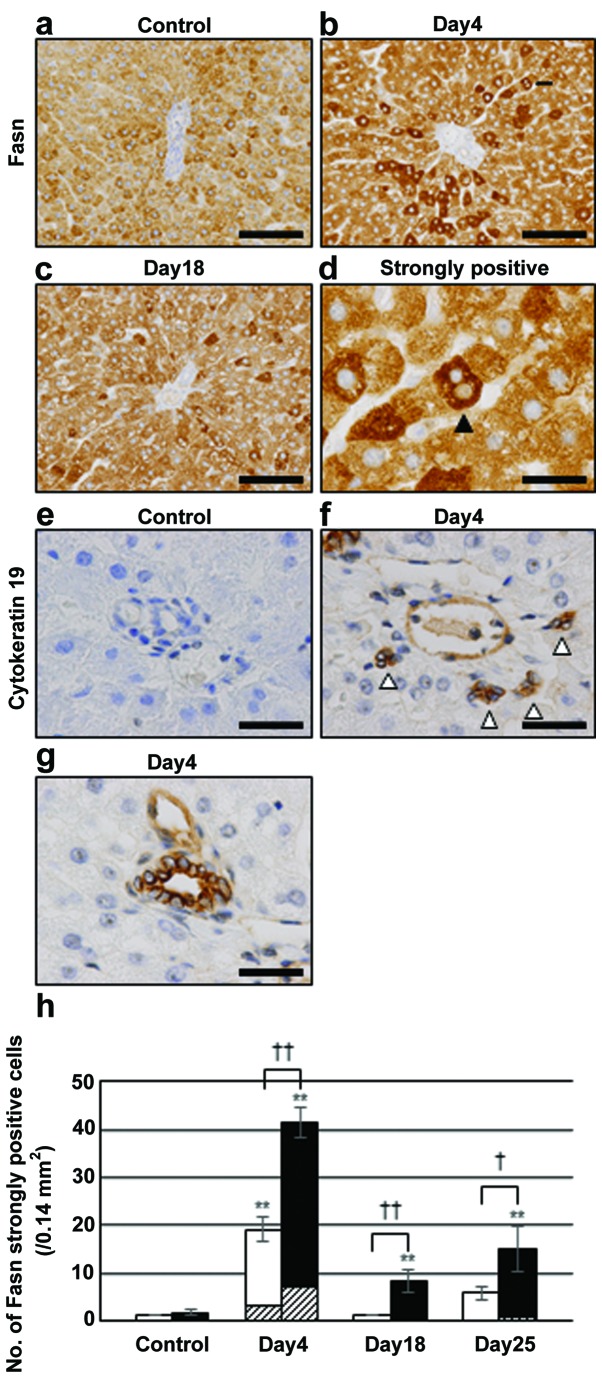
(a–d) Immunostaining for Fasn, and (e–g) cytokeratin 19 in (a and e) control and irinotecan group (b, d, f and g) on day 4, and the number of (h) Fasn-strongly-positive hepatocytes. (b) An arrow and (d) a closed arrowhead indicate cells accompanying two nuclei, and (f) open arrowheads show cytokeratin 19-positive cells forming clusters. The data shown are from a representative preparation set and are similar to results obtained in two other sets. (a–c) Scale bars 100 *μ*m, and (d–g) 25 *μ*m. (h) The number of Fasn-strongly-positive hepatocytes. Open bars indicate the number around the CV and closed bars around the BD. Hatched bars represent the number of cells with two nuclei. Data are the mean ± SD from 3 rats. These cells were quantified from five microscope fields (0.14 mm^2^/field) for each rat. ^**^p<0.01 vs. control (two-tailed Student’s t-test). ^†^p<0.05 and ^††^p<0.01 between the values for CV and the BD (two-tailed Student’s t-test). Fasn, fatty acid synthase; CV, central vein; BD, bile ducts.

Immunostaining was performed to examine whether Fasn protein levels also increased. Most hepatocytes in the controls were weakly positive for Fasn (Fig. 3a), whereas strongly positive hepatocytes appeared around the BD on day 4 in the irinotecan group (Fig. 3b) and frequently included cells with two nuclei (Fig. 3b). Fasn-strongly-positive cells were decreased on day 18 (Fig. 3c). Polykaryonic cells were also observed in Fasn-weakly-positive hepatocytes on day 4, but at a lower frequency. The size of polykaryonic cells heavily stained for Fasn was smaller than that of weakly stained cells (Fig. 3d).

### Activation of liver progenitor cells

Immunohistochemistry was performed for cytokeratin 19 in order to examine alterations in liver progenitor cells. Although a positive reaction was not obtained in the control (Fig. 3e), liver progenitor cells that formed clusters around portal veins (Fig. 3f) were detected on day 4 in the irinotecan group. In addition, bile duct epithelial cells also showed positive reaction (Fig. 3g). These results demonstrated the activation of liver progenitor cells following the administration of irinotecan, whereas their morphology and localization differed from those of Fasn-strongly-positive hepatocytes (Fig. 3b).

### Relationship between Fasn-strongly-positive hepatocytes and CA3-positive hepatocytes with vacuoles

To examine the relationship between Fasn-strongly-positive cells and CA3-positive hepatocytes accompanying intracellular vacuoles, the localization of these cells was compared. Both cell types were frequently distributed around the BD, but their locations differed (Figs. 3b and 2c). The numbers of Fasn-strongly-positive hepatocytes around the BD increased to 41.3±3.1 on day 4, and then decreased to 8.3±2.5 on day 18 (Fig. 3h). Approximately 17% of Fasn-strongly-positive hepatocytes possessed two nuclei (Fig. 3h) on day 4. The number of CA3-positive hepatocytes accompanying intracellular vacuoles around BD was 13.7±0.6 on day 4, and this increased to 43.0±5.3 on day 18 (Fig. 2f). Thus, the magnitude of the decrease in the number of Fasn-strongly-positive hepatocytes between days 4 and 18 was similar to that of the increase in the number of CA3-positive hepatocytes accompanying intracellular vacuoles. Similar results were also observed in the values around the CV between days 4 and 18 (Figs. 3h and 2f).

### Loss of Kupffer cell marker proteins

**Figure 4:**
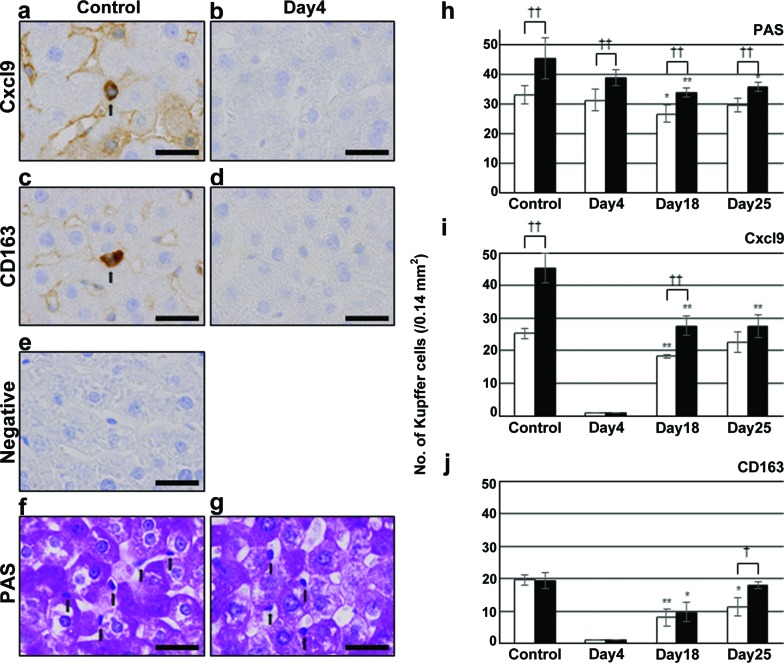
Immunostaining for (a and b) Cxcl9, (c and d) CD163 and (f and g) PAS staining in (a, c, e and f) control and irinotecan group on (b, d and g) day 4. Liver sections of control group were also stained with (e) non-immune γ-globulin. (a, c, f and g) Arrows indicate positive-Kupffer cells. The data shown are from a representative preparation set and are similar to results obtained in two other sets. Scale bars represent 25 *μ*m. The number of Kupffer cells counted with (h) PAS staining and immunostaining for (i) Cxcl9 and (j) CD163 in control and irinotecan group on days 4, 18 and 25. Open bars indicate values around the CV and closed bars around the BD. Data are the mean ± SD from 3 rats. These cells in liver sections were from five microscope fields (0.14 mm^2^/field) for each rat. ^*^p<0.05 vs. control; ^**^p<0.01 vs. control (two-tailed Student’s t-test). ^†^p<0.05 between values around the CV and those around the BD; ^††^p<0.01 between values for the CV and for the BD (two-tailed Student’s t-test). Cxcl9, chemokine (C-X-C motif) ligand 9; PAS, periodic acid-Schiff; CV, central vein; BD, bile ducts.

To examine the effects of irinotecan on Kupffer cells, the expression of their marker proteins, Cxcl9 and CD163, was evaluated by immunohistochemistry. As shown in Fig. 4a, Kupffer and sinusoidal endothelial cells were positive for the anti-Cxcl9 antibody in the control, but were negative on day 4 (Fig. 4b), and became positive again on days 18 and 25 (data not shown). Immunostaining for CD163 and CD68 was also negative on day 4 (Fig. 4c and d for CD163, and data not shown for CD68). Non-immune γ-globulin, instead of the primary antibodies, was also negative (Fig. 4e). On the other hand, PAS staining revealed the presence of Kupffer cells on day 4 (Fig. 4g) as well as in the control (Fig. 4f). Therefore, the results of immunostaining indicated the loss of Kupffer cell markers, but the cells were still present on day 4. The numbers of Kupffer cells detected by PAS staining were higher around the BD than the CV at each time point (Fig. 4h). The number of Cxcl9-positive Kupffer cells around the BD was also higher than that around the CV in the control (Fig. 4i), whereas that of CD163-positive cells was similar between the two areas (Fig. 4j). Cxcl9-positive Kupffer cells decreased to 0 on day 4, and then increased to 27.7±3.1 around BD on day 18, but was still lower than the value obtained in the control (Fig. 4i). CD163-positive Kupffer cells also decreased to 0 on day 4, and then increased to 9.7±3.1 around BD on day 18 (Fig. 4j).

## Discussion

Although H&E staining of liver sections and immunohistochemistry for CA3 revealed no significant changes on day 4, panacinar intrahepatocyte vacuoles were present on day 18, and had disappeared by day 25. Microarray and RT-PCR analyses showed that the expression of genes involved in the synthesis of cholesterol and fatty acids was upregulated on day 4. Thus, genes involved in the synthesis of fatty acids were upregulated on day 4, and hepatic steatosis appeared on day 18. Immunostaining detected the appearance of Fasn-strongly-positive hepatocytes on day 4. Some cells possessed two nuclei and were small in size. Since the expression of Fasn is reported in proliferating cells (27), the results of the present study suggested that Fasn-strongly-positive hepatocytes were mitotic. The magnitude of the decrease in the number of Fasn-strongly-positive hepatocytes between days 4 and 18 was similar to that of the increase in the number of CA3-positive hepatocytes accompanying intracellular vacuoles, suggesting that the former cells changed into the latter cells. The degree of steatosis was more prominent around the BV than the CV and this may have been related to the preferential localization of Fasn-strongly-positive hepatocytes.

Irinotecan has been identified as an inhibitor of topoisomerase 1, an enzyme involved in DNA replication (10); therefore, withdrawal of the drug may have induced cell proliferation. However, microarray analysis revealed that the expression of genes involved in cell proliferation was downregulated on day 4. Moreover, liver progenitor cells, known as oval cells in rodents, were previously shown to proliferate around the portal vein in response to suppression of hepatocyte proliferation by some agents, to form ductular structures and then expanded into liver parenchyma (28,29). Activation of progenitor cells was demonstrated by immunohistochemistry for cytokeratin 19 in the present study. A previous study reported that the activation of liver progenitor cells was correlated with progression toward NASH (30). This raises a possibility that activated progenitor cells may differentiate into Fasn-strongly-positive hepatocytes. However, both cells were found to be morphologically different and cells that shared the properties of both cell types were not detected. Thus, it is unlikely that such progenitor cells were directly changed into Fasn-strongly-positive cells.

Kupffer cells were previously shown to promote steatohepatitis by enhancing hepatic lipid accumulation through various inflammatory mediators, including specific cytokines (31). However, Kupffer cell dysfunction is also known to induce nonalcoholic fatty liver disease (32). Immunostaining for Cxcl9 and CD163 indicated that Kupffer cell marker proteins were absent on day 4, even though Kupffer cells were present. Such protein loss appeared to be specific to Kupffer cells, since CA3 and Fasn proteins were retained in hepatocytes. The expression of *Eif2ak4* gene, which mediates stress responses that suppress global protein synthesis (33) was upregulated on day 4. Kupffer cell dysfunction may be partly involved in the appearance of Fasn-strongly-positive hepatocytes. The number and localization of Cxcl9-positive Kupffer cells differed from those of CD163-positive Kupffer cells. As CD163 is a marker for M2 macrophages (34), CD163-positive cells may belong to M2 Kupffer cells (35), while Cxcl9-positive cells seem to belong to the M1 cells.

In summary, hepatic steatosis was induced by irinotecan on day 18 and preceded by Fasn-strongly-positive hepatocytes and the activation of liver progenitor cells. The former cells are suggested to change into lipid-accumulating hepatocytes.

## Figures and Tables

**Table I:** RT-PCR primer sequences.

Gene	5′-Primer (5′–3′)	3′-Primer (5′–3′)
*Hmgcr*	GCCCAAAATTTGAAGAGGACGTG	CCGAGAAAGCTCTAGGACCAGGG
*Idi1*	TGAAAACATTGACAAAGGACTAATACATCGAG	TCATTTCATTTAGATCAACCTCTTCCAAGG
*Sqle*	TCCAAGAGGCGCAGAAAAGAAGTC	TGTATCTCCAAGGCCCAGCTCTC
*Cyp7a1*	GAATTGCCGTGTTGGTGAGCTG	GCTTCTGTGTCCAAATGCCTTCG
*Abcg5*	TTCAGCGTCAGCAACCGTGTC	TGTCAGGACTGCCTCTACCTTCTTGTC
*Abcb11*	TTTTCCAGAGGCAGCTATCG	ATGGCTGCACTCAAAGATCC
*Slc10a1*	AGGCATGATCATCACCTTCC	AAGTGGCCCAATGACTTCAG
*Ugt2b*	ACATTTTACAGTGAGATTTTGGGAAGGC	AGGATGTCATTCTGCGGGAGC
*Fasn*	TCCCAGGTCTTGCCGTGC	GCGGATGCCTAGGATGTGTGC
*Acss2*	GCTTTTTACTGGGAGGGCAATGAG	CCTTCTCTCGGCACTTCTCCAAG
*Acsm5*	GCTTGTATGCGAACAGGTGTGGTC	CCTTCCACTGGCCACAAAACC
*Acsl5*	ATTGAGGGAGGAGCACGGAGAG	TCAGCTCTGTTGATGACATAGATGATGG
*Elovl6*	CTCTTGCGGTCTTCAGTATATTCGGTG	TCCTCAGAATGATGAATATCGTATCACCTAGTTC
*Egr1*	AGCACCTGACCACAGAGTCCTTTTC	ACGGCACGGCACAGCTC
*Cxcl9*	TCGAGGAACCCTAGTGATAAGGAATCAG	TTTGCTTTTTCTTTTGGCTGATCTTTTTC
*Ly6c*	GTGTGCAGAAAGAGCTCAGGGC	TGTCCGTCTTACAGAGCCCTCTACAG
*β-actin*	GTACCACTGGCATTGTGATG	ATCTTCATGGTGCTAGGAGC

Fasn, fatty acid synthase.

**Table II:** The results of microarray analysis in control and irinotecan group at days 4, 18 and 25.

Cellular function and gene name	Gene symbol	Signal			
		Control	Day 4	Day 18	Day 25
Cholesterol synthesis					
Citrate					
ATP-citrate lyase	*Acly*	2,096	5,121	2,511	1,700
Acetyl-CoA					
Acetoacetyl-CoA synthetase	*Aacs*	91	1,462	309	226
HMG-CoA synthase 1	*Hmgcs1*	2,691	8,433	4,088	5,586
HMG-CoA reductase	*Hmgcr*	1,001	5,012	1,878	1,276
Mevalonate					
Mevalonate pyrophosphate decarboxylase	*Mvd*	265	1578	495	478
Isopentenyl-diphosphate Δ-isomerase 1	*Idi1*	488	2467	675	933
Squalene					
Squalene epoxidase	*Sqle*	362	2,980	980	870
Lanosterol					
Cytochrome P450, family 51	*Cyp51*	3,430	8,418	4,975	4,194
7-Dehydrocholesterol reductase	*Dhcr7*	1,408	4,582	1,912	1,996
Bile acid synthesis					
Cytochrome P450, family 7, subfamily a, polypeptide 1	*Cyp7a1*	1,242	7,299	1,671	1,250
Cytochrome P450, family 27, subfamily a, polypeptide 1	*Cyp27a1*	2,228	5,076	2,781	3,078
Cholesterol and bile acid export					
Bile canaliculus					
ATP-binding cassette sub-family G member 5	*Abcg5*	515	1,345	827	542
ATP-binding cassette sub-family G member 8	*Abcg8*	222	794	476	169
Sinusoid					
ATP-binding cassette, sub-family C (CFTR/MRP), member 3	*Abcc3*	230	1,583	595	297
Bile acid conjugation					
UDP glucuronosyltransferase 2 family, polypeptide A3	*Ugt2a3*	1,852	3,972	2,538	2,912
UDP glucuronosyltransferase 2 family, polypeptide B	*Ugt2b*	20	6,163	4,034	4,731
Fatty acid synthesis					
Fatty acid synthase	*Fasn*	2,096	5,140	2,650	1,687
Elongation of long chain fatty acid member 6	*Elovl6*	461	5,188	500	571
Acyl-CoA synthetase					
Acyl-CoA synthetase short-chain family member 2	*Acss2*	1,367	6,140	2,190	1,892
Acyl-CoA synthetase medium-chain family member 2	*Acsm2*	251	1,199	820	213
Acyl-CoA synthetase long-chain family member 5	*Acsl5*	3,079	5,723	4,308	3,181
Transcription factor					
Sterol regulatory element-binding factor 2	*Srebf2*	1,068	1,826	1,365	1,358
Insulin induced gene 1	*Insig1*	1,734	6,462	3,397	3,819
SREBP cleavage-activating protein	*Scap*	888	1,779	1,014	900
Early growth response 1	*Egr1*	629	761	2,932	1,308
Nuclear receptor subfamily 1, group I, member 3	*Nr1i3*	563	792	1,469	1,210
DNA					
Cell cycle					
Cyclin B1	*Ccnb1*	99	48	267	195
Cyclin-dependent kinase inhibitor 3	*Cdkn3*	128	50	271	172
DNA replication					
Ribonucleotide reductase M2	*Rrm2*	156	60	378	534
Topoisomerase (DNA) IIα	*Top2a*	154	71	391	376
Mitosis					
Cell division cycle 20 homolog (*S. cerevisiae*)	*Cdc20*	165	103	293	343
Cytoskeleton associated protein 2	*Ckap2*	77	33	175	154
ER stress					
Eukaryotic translation initiation factor 2α kinase 4	*Eif2ak4*	314	549	402	337
Kupffer cells					
CD68 molecule	*Cd68*	612	722	654	761
CD163 molecule	*Cd163*	747	831	713	691
Mannose receptor, C type 1	*Mrc1*	1,383	1,408	1,317	1,402
Chemokine (C-X-C motif) ligand 1	*Cxcl1*	251	710	540	371
Chemokine (C-X-C motif) ligand 9	*Cxcl9*	1,118	282	423	923
Monocytes					
Ly6-C antigen	*Ly6c*	219	144	500	560
Sinusoids					
CD34 molecule	*Cd34*	100	93	98	101
Stellate cells					
Collagen, type I, α1	*Col1a1*	320	395	582	397
Collagen, type I, α2	*Col1a2*	426	532	610	405
Desmin	*Des*	133	171	116	108
Liver progenitor cells					
Cytokeratin 19	*Krt19*	111	224	155	115
Epithelial cell adhesion molecule	*Epcam*	236	545	242	185

**Table III:** The mRNA by RT-PCR evaluation for cholesterol and fatty acid metabolism in control and irinotecan group.

Cellular function and gene name	Gene symbol	Signal			
		Control	Day 4	Day 18	Day 25
Cholesterol synthesis					
HMG-CoA reductase	*Hmgcr*	1	10.14	1.07	1.97
Isopentenyl-diphosphate Δ-isomerase 1	*Idi1*	1	9.9	1.07	0.92
Squalene epoxidase	*Sqle*	1	55.19	12.54	6.7
Bile acid synthesis					
Cytochrome P450, family 7, subfamily a, polypeptide 1	*Cyp7a1*	1	7.73	1.91	0.59
Cholesterol and bile acid export					
ATP-binding cassette, sub-family G member 5	*Abcg5*	1	1.17	1.88	0.55
ATP-binding cassette, sub-family B (MDR/TAP), member 11	*Abcb11*	1	1.07	1.02	1.11
Solute carrier family 10 (sodium/bile acid co-transporter family), member 1	*Slc10a1*	1	1.24	0.87	1.84
Bile acid conjugation					
UDP glucuronosyltransferase 2 family, polypeptide B	*Ugt2b*	1	7,362	3,310	2,392
Fatty acid synthesis					
Fatty acid synthase	*Fasn*	1	3.58	3.85	0.81
Acyl-CoA synthetase short-chain family member 2	*Acss2*	1	12.11	6.9	2.14
Acyl-CoA synthetase medium-chain family member 5	*Acsm5*	1	11.94	22.49	6.03
Acyl-CoA synthetase long-chain family member 5	*Acsl5*	1	5.53	6.74	1.99
Elongation of long chain fatty acid member 6	*Elovl6*	1	20.23	1.19	0.55
Transcription factor					
Early growth response 1	*Egr1*	1	3.55	23.32	6.16
Kupffer cells					
Chemokine (C-X-C motif) ligand 9	*Cxcl9*	1	0.2	0.34	0.51
Monocytes					
Ly6-C antigen	*Ly6c*	1	0.55	1.26	1.62

mRNA levels in the irinotecan group are expressed relative to the values of individual mRNAs in the control group.

## Abbreviations

H&E: hematoxylin and eosin
RT-PCR: real-time polymerase chain reaction
Fasn: fatty acid synthase
CA3: carbonic anhydrase 3
CRLM: colorectal cancer liver metastasis
CPT-11: irinotecan
NASH: nonalcoholic steatohepatitis
Cxcl9: chemokine (C-X-C motif) ligand 9
NAFLD: nonalcoholic fatty liver disease
CV: central veins
BD: bile ducts
Srebp2: sterol regulatory element-binding protein 2
Insig1: insulin induced gene 1
Scap: SREBP cleavage-activating protein
Eif2ak4: eukaryotic translation initiation factor 2α kinase 4
PAS: periodic acid-Schiff
Egr1: early growth response 1
Nr1i3: nuclear receptor subfamily 1, group I, member 3
CASH: chemotherapy-associated steatohepatitis
